# A needle in the haystack: An unusual case presentation of ganglioneuroblastoma at a tertiary care center in Coastal Karnataka

**DOI:** 10.1002/ccr3.8149

**Published:** 2023-11-22

**Authors:** Shalini Radhakrishnan, Ranjitha Rao, Harsha Prasada Lashkari, Hema Kini, Jyoti Ramnath Kini, Vatsala Basavaraju Kudurugundi, Vanishree Ashok, Chaithra Venkataramana Gowthuvalli

**Affiliations:** ^1^ Department of Pathology Kasturba Medical College Mangalore, Manipal Academy of Higher Education Manipal India; ^2^ Department of Paediatric Haematology and Oncology Kasturba Medical College Mangalore, Manipal Academy of Higher Education Manipal India; ^3^ Kasturba Medical College Mangalore, Jyothi Circle Mangalore India

**Keywords:** atypical presentation, case reports, ganglioneuroblastoma, histopathological examination, morphological diversity, neuroblastic tumors

## Abstract

**Key Clinical Message:**

This case report highlights the importance of recognizing and accurately diagnosing ganglioneuroblastoma, an uncommon variant of neuroblastic tumors in children. Ganglioneuroblastomas have diverse clinical and morphological presentations, and histopathological examination is paramount in guiding treatment decisions, especially in cases with ambiguous symptoms. Early detection is crucial, as the prognosis varies significantly based on the subtype and the presence of metastatic disease. Clinicians should maintain a high index of suspicion and utilize radiological examinations to promptly identify and treat these tumors.

**Abstract:**

Children are frequently affected by neuroblastic tumors, which grow from the sympathoadrenal lineage of the neural crest during its development. However, intermixed ganglioneuroblastomas are far less common within the same tumor spectrum, the diagnosis of which could become challenging amidst an unusual presentation. In our case report, we present a 4‐year‐old boy who had complaints of fever and difficulty in walking, with a supra‐renal mass on ultrasound, which was diagnosed as ganglioneuroblastoma‐intermixed type on histopathological examination. This report aims to contribute to the understanding of the diverse clinical and morphological spectrum of ganglioneuroblastomas and the importance of multidisciplinary collaboration and histopathological examination to enhance decision‐making in such ambiguous scenarios.

## Graphical abstract

Microscopy and immunohistochemical findings suggestive of Ganglioneuroblastoma Schwannian stroma rich type.

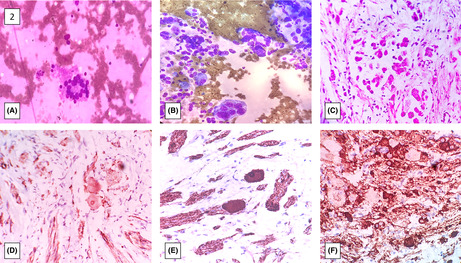

## 1 INTRODUCTION

After brain tumors and leukemias, neuroblastic tumors are the third most common childhood neoplasm.^1^ However, ganglioneuroblastoma is an uncommon subtype among peripheral neuroblastic tumors. Each year, fewer than five cases per million children are believed to occur. There is an extensive variation in the clinical manifestations of these tumors. Patients could be completely asymptomatic, or they could have symptoms that are vague, connected to potential metastases, or indicative of paraneoplastic syndromes. Ganglioneuroblastomas usually have a favorable prognosis. Tumor resection is frequently used to treat patients instead of cytotoxic therapy. However, the “favorable histology” has not been supported by anecdotal instances of ganglioneuroblastomas with violent behavior that have been published. Particularly in the context of metastatic disease, little is known about the molecular characteristics, clinical prognosis, and behavior of primary ganglioneuroblastoma.^2^ Hence, it may be challenging to diagnose this malignancy amidst an atypical presentation. We present the case of a 4‐year‐old boy who was diagnosed with ganglioneuroblastoma on histopathological examination.

## 2 CASE PRESENTATION

A 4‐year‐old boy presented with complaints of intermittent fever and difficulty in walking for one and a half months. Occasional headaches were also noted. There was no history suggestive of trauma, tingling, numbness, rashes, or vomiting. Developmental milestones were appropriate for age. A general and systemic examination was done, which was unremarkable. A head‐to‐toe examination revealed left sacroiliac joint tenderness. Laboratory investigations revealed elevated C‐reactive protein (CRP) [41.9 mg/dL], lactate dehydrogenase (LDH) [390 IU/L], and mildly elevated erythrocyte sedimentation rate (ESR) [46 mm/hr]. Magnetic resonance imaging (MRI) of the hip joint showed focal marrow edema in the intertrochanteric region of the femur.

Differentials of osteomyelitis, juvenile idiopathic arthritis (JIA), and malignancies such as leukemias were considered. A peripheral blood smear was performed, which showed mild leucocytosis. ASO titres and rheumatoid factor (RF) were analyzed and determined to be negative. An anti‐nuclear antibody (ANA) profile was also performed, which turned out to be negative.

**FIGURE 1:**
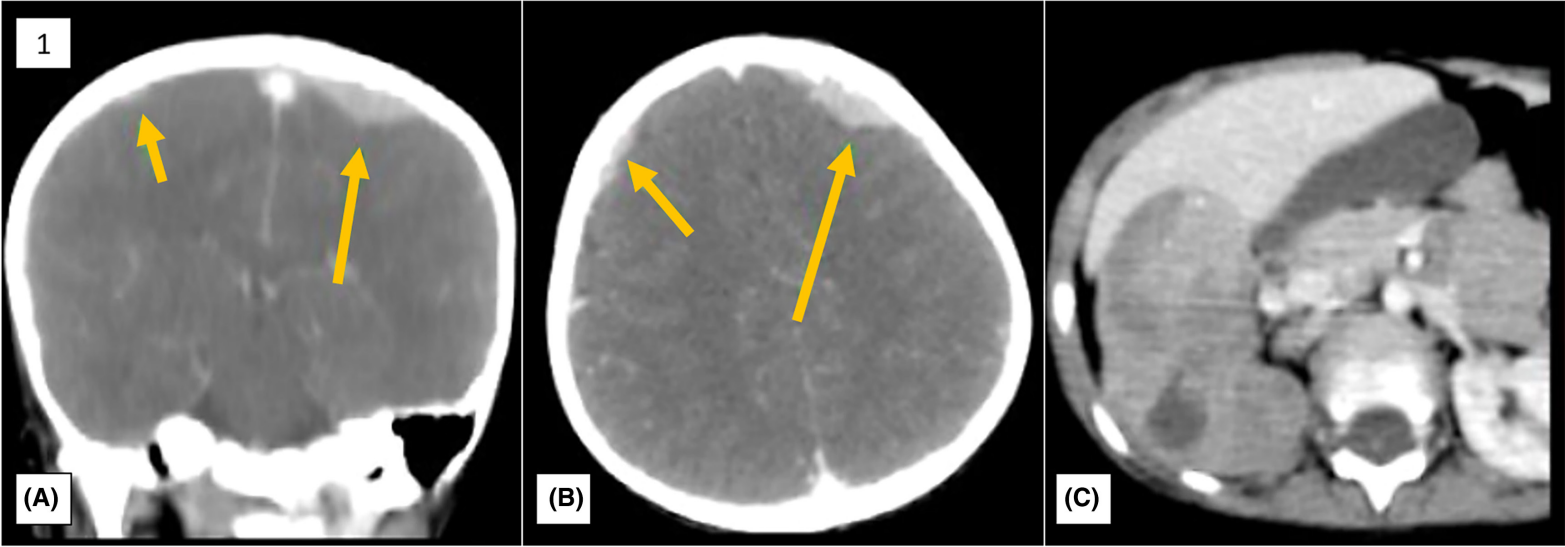
F/18 FDG PET/CT scan from head to mid‐thigh: FDG uptake increased in bilateral frontal and parietal regions and right suprarenal region.

In view of increased LDH, a bone marrow aspiration was performed, which was a dilute marrow showing many small‐ to medium‐sized cells with hyperchromatic nuclei, indiscernible to small amounts of cytoplasm, and vague cytoplasmic borders, arranged in singles, dense clusters, and rosettes with fibrillary eosinophilic material in the lumen. Abdominal and pelvic ultrasounds were performed later, which showed a large lobulated hypoechoic space‐occupying lesion (SOL) with multiple specks of calcifications measuring 8.7 × 5.0 × 5.9 cm in the right suprarenal region. A diagnosis of neuroblastoma was given, and pathological correlation was advised. USG‐guided fine needle aspiration cytology (FNAC) was performed from the SOL, which showed tumor cells arranged in small clusters having coarse chromatin and scant cytoplasm, with the presence of occasional true rosettes. A diagnosis of a small, round blue‐cell tumor suggestive of neuroblastoma was given. In addition, positron emission tomography and computed tomography (PET CT) were performed, which showed fludeoxyglucose F18 (FDG)‐avid, fairly homogeneous dural‐based lesions involving bilateral parietal and frontal regions. A maximum thickness of 1 cm was seen in the left posterosuperior parietal region. The rest of the neural parenchyma was unremarkable. The right suprarenal region also showed an FDG‐avid large lobulated heterogeneously enhancing soft tissue density mass lesion along with some necrotic areas. Fludeoxyglucose uptake was increased in a few enlarged upper aortocaval and retrocaval lymph nodes. Abnormal FDG uptake in the bone marrow at multiple sites was also observed. A diagnosis of malignant etiology indicative of neuroblastoma with metastasis was given, and a biopsy correlation was suggested. (Figure 1).

**FIGURE 2:**
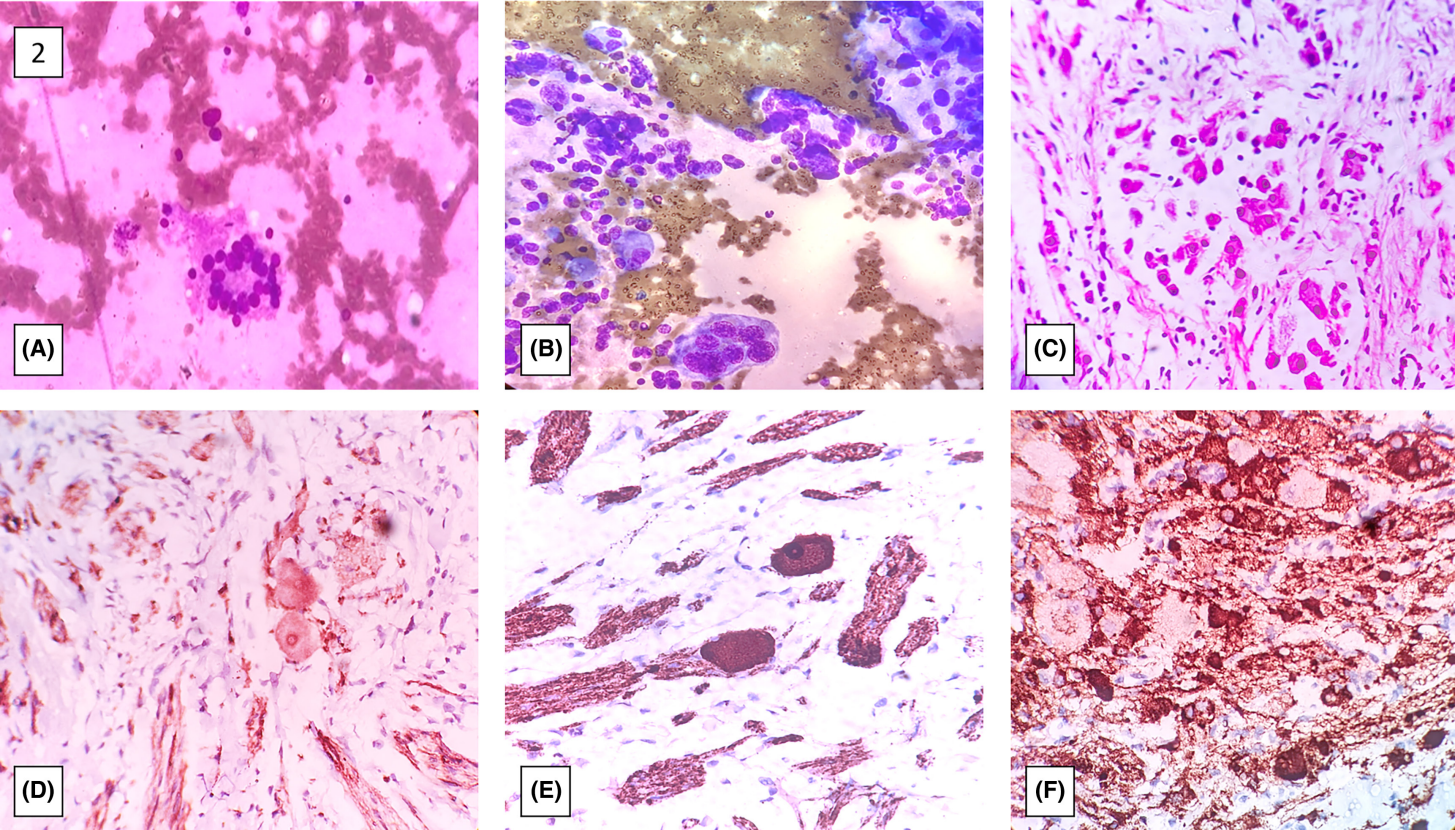
(A) Bone Marrow Aspiration Smear: Small round blue tumour cells arranged in a rosette. (B) Fine Needle Aspiration Cytology from the suprarenal mass: Tumour cells seen arranged in a rosette and singly scattered. (C) Core biopsy from the suprarenal mass: Tumour cells with eccentric nuclei scattered in singles and small clusters. (D–F) Immunoprofile of the core biopsy from the suprarenal mass: (D). Positive cytoplasmic staining of GFAP. (E) Granular cytoplasmic staining of chromogranin. (F) Positive membranous staining of synaptophysin.

To confirm, a core biopsy was sampled from the SOL under USG guidance, which showed linear tissue cores with tumor cells that were predominantly large and oval with small eccentric nuclei and fibrillary cytoplasm, arranged in nests and singles. A few small round cells with hyperchromatic nuclei were also observed in small clusters along with the large cells. The stroma surrounding the tumor cells was fibro‐collagenous in nature with focal fibrillary stroma and certain foci showed calcifications as well. An immunohistochemical analysis was performed for further evaluation; the results revealed positivity for chromogranin, synaptophysin, glial fibrillary acidic protein (GFAP), and neuron‐specific enolase (NSE). The ganglion cells had taken up GFAP, with the neural filaments and neuroblasts taking up chromogranin, synaptophysin, and NSE. Considering all the aforementioned findings, a histopathological diagnosis of ganglioneuroblastoma‐intermixed type (Schwannian stroma rich) was given. However, the likelihood of ganglioneuroblastoma‐nodular type (composite areas of well‐differentiated/mature tissue alternating with immature tumor tissue) was also taken into account given the already metastatic bone marrow disease and the cytology of the same mass lesion showing predominantly small round cells. (Figure 2) For risk stratification, a MYCN gene amplification assay was performed, which turned out to be negative.

The patient's post‐diagnosis course was closely monitored to assess treatment response and disease progression. The child was started on radiotherapy, and routine follow‐ups were scheduled, which showed no improvement. Subsequent imaging performed showed evidence of metastasis to the brain with no complete remission of the disease. Unfortunately, the child was lost for follow‐up later.

## 3 DISCUSSION

A variety of neuroblastic tumors arising from primitive sympathetic ganglion cells include neuroblastoma (NB), ganglioneuroblastoma (GNB), and ganglioneuroma (GN); additionally, these solid extracranial tumors are frequently found in children. These tumors may be identified by their level of cellular maturation and differentiation, as well as by the proportions of Schwann cells (Schwannian‐blasts and mature Schwann cells) and neural‐type cells (primitive neuroblasts, maturing neuroblasts, and ganglion cells) in each one of them.^3^ According to the International Neuroblastoma Pathology Classification (INPC) system, NB is malignant, GNB has intermediate malignant potential, and GN is regarded as a benign tumor.^4^

Less than five cases per million children are reported to occur annually.^5^ Most of the children affected are below 5 years of age, with the disease being slightly more common in boys than in girls.^1^ In contrast to NB, which more frequently affects newborns and infants, GNB is uncommon in the primary setting and frequently affects older children. The tumor is thought to be in a transitional stage where it is progressing towards complete differentiation from NB to GN, a process that is incomplete as shown by occasional microscopic pockets of NB.^6^

Clinical signs and symptoms are comparable in NB and GNB, which include abdominal distension and constipation caused by an abdominal mass, respiratory distress brought on by a mediastinal mass, and neurological signs and symptoms brought on by paraspinal tumors that have spread into the spinal canal. If metastasized, the clinical features could range from generalized lymphadenopathy to bone pain with racoon eyes.^1^ Bone metastasis is extremely common, with patients presenting with complaints such as limping and unexplained irritability (Hutchinson's syndrome).^3^ According to a study by He W et al.,^4^ at the time of the initial diagnosis, metastases were observed to appear in approximately 60% of NB patients. Paraneoplastic syndrome as a result of the release of vasoactive intestinal peptide (VIP), known as Verner‐Morrison syndrome, is also documented in GNB.^1^ As a result, the clinical manifestations of neuroblastic tumors are complex, and health care professionals should be aware of both the conventional clinical manifestations as well as atypical manifestations, particularly those brought about by disseminated tumors.

The most common locations for GNB are the adrenal gland (35%), retroperitoneum ganglia (30%–35%), posterior mediastinum (20%), and pelvis (2%–3%), but it can also develop in the brain and the cervical area.

Radiological tests such as USG and CT/MRI can locate these mass lesions. Since these tumors frequently result in aberrant catecholamine synthesis, secretion, or catabolism, laboratory testing includes a measurement of catecholamines and their metabolites in the blood and urine. There are two approaches to establishing the diagnosis of neuroblastic tumors: either by histological examination of the main tumor or by finding both tumor cells during a puncture biopsy of the bone marrow and a rise in catecholamines or urine catecholaminergic metabolites.^7^

Due to their wide range of differentiation in terms of morphology, small biopsy samples brought on by the rising use of core needle biopsies (often image‐guided), or FNACs, and morphological overlap with other mesenchymal tumors, neuroblastic tumors present significant diagnostic challenges.^8^ The IPNC defines two subcategories of GNB: the intermixed subtype (GNBi), which is Schwannian stroma rich, and the nodular subtype (GNBn), which could be composite, Schwannian stroma rich, or Schwannian stroma poor. The INPC system has incorporated four tumor categories under two distinct prognostic groups: unfavorable histology (UH) and favorable histology (FH). By incorporating the approach created by Shimada et al., the INPC was the first to designate tumor groups using histologic indications of both grades of neuroblastic differentiation and Schwannian stromal development.^8^, ^9^

On macroscopic evaluation, GNBs are soft, circumscribed, grayish‐tanned masses. They may or may not have foci of hemorrhage, calcifications, and necrosis.^1^ The microscopic nests of neuroblastic cells that make up the GNBi subtype are dispersed randomly throughout the ganglioneuromatous tissue. By definition, the tumor's ganglioneuromatous component must account for more than half of its overall volume.^1^, ^8^, ^10^

Small round cell tumors comprise neuroblastoma and a few sarcoma subtypes such as alveolar rhabdomyosarcoma, melanoma, carcinoma, and lymphoma/leukemia. These tumors are composed of a monotonous proliferation of small, round cells with scant cytoplasm. Due to the substantial morphological overlap among the aforementioned tumors, diagnosis may be difficult. A crucial role in the diagnosis of soft tissue malignancies is played by immunohistochemistry. It is used to exclude non‐sarcomatous entities and determine which line of mesenchymal differentiation (if any) the tumor cells display to diagnose small round cell tumors.^1^, ^7^, ^8^, ^11^ In neuroblastic tumors, immunohistochemical staining with antibodies such as neurofilament protein (NFP), synaptophysin, chromogranin, and S‐100 is typically positive. Compared to neuroblastic tumors, alveolar rhabdomyosarcomas have more nuclear pleomorphism, a greater amount of cytoplasm, and are positive for muscle‐specific markers such as desmin, myogenin, and MyoD. A desmoplastic stroma with nests of small round cells and immuno‐positivity for cytokeratin, desmin (a dot‐like pattern), and WT1 are both seen in desmoplastic small round blue cell tumors (DSRCTs). Extraskeletal Ewings Sarcoma is typically found in older patients and shows diffuse membrane positivity for CD99. Undifferentiated small round‐cell sarcomas have delicate vasculature, a variable myxoid stroma, and primitive round‐to‐spindle cells organized in nests, sheets, or fascicular growth patterns, which are positive for the immunohistochemical stains SATB2 and cyclin D1. Leukocyte Common Antigen (LCA), CD3, CD20, and Tdt are only a few of the lineage‐specific hematopoietic markers that lymphomas and leukemias exhibit but neuroblastic tumors do not.^3^

A significant oncogenic driver of NB is thought to be the MYCN oncogene, which is found on chromosome 2p24.3. It is seen in approximately 20%–25% of cases of neuroblastoma and correlates with high‐risk disease and poor prognosis. GNBs usually have a favorable prognosis because of their histology and absence of MYCN gene amplification.^10^ However, there have been isolated instances of GNBi with aggressive behavior that is at odds with the “favorable histology.”.^6^

Stage, histologic classification, chromosomal status (ploidy and 11q aberration), genetics (MYCN amplification), and patient age are all included in the most recent International Neuroblastoma Risk Group (INRG) classification system, which was adopted in 2009. Localized (stage L1), regional (stage L2), metastatic (stage L3), and “special stage” (stage MS) are a quick overview of the INRG classification system.^6^

Treatment modalities include complete surgical resection of the tumor. Chemotherapy is not advised due to its poor response, increased toxicity, inadequate tumor volume reduction, and other factors. These malignancies are known to exhibit spontaneous regression, tumor maturation, and aggressive progression resistant to therapy.^3^, ^9^, ^10^, ^12^, ^13^

Subsets of patients with peripheral neuroblastic tumors are being identified more frequently based on spontaneous regression or differentiation and excellent overall outcomes, such as those with low‐ and intermediate‐risk NB and favorable clinical and biological characteristics (e.g., infants with small perinatal adrenal masses or those with favorable histology and absence of segmental aberrations). It is recommended to perform routine surveillance with chest X‐rays and MRI scans for mediastinal tumors for at least 2 years, as well as ultrasound or MRI scans for abdominal or paraspinal tumors.^5^, ^9^, ^12^, ^13^, ^14^

The most clinically significant prognostic variables in prior research have been identified as patient age, tumor stage, histology, tumor grade, MYCN oncogene status, chromosomal 11q status, and DNA ploidy. Infants have a higher rate of survival than older children. Comparing patients with confirmed GNBi to those with NB, patients with NB, however, are more likely to show unfavorable prognostic factors than patients with GNBi.^4^ In a study conducted by Nezami B et al.,^6^ GNBi was found to be associated with a favorable prognosis with 100% progression‐free survival (PFS) and overall survival (OS) when detected at initial presentation in patients without metastatic disease.

## 4 CONCLUSION

Ganglioneuroblastomas are rare when compared to neuroblastomas. Ganglioneuroblastoma‐intermixed type (Schwannian stroma‐rich) without metastatic disease at presentation will have a good prognosis. However, it may cause an undue delay in diagnosis if it exhibits unique symptoms as a result of metastatic disease. A high index of suspicion and radiological examination must be carried out to rule out a mass lesion in doubtful cases.

## AUTHOR CONTRIBUTIONS

**Shalini Radhakrishnan:** Conceptualization; data curation; formal analysis; investigation; writing – original draft; writing – review and editing. **Ranjitha Rao:** Formal analysis; methodology; project administration; supervision; validation; writing – review and editing. **Harsha Prasada Lashkari:** Data curation; formal analysis; investigation; methodology; supervision; validation; visualization. **Hema Kini:** Investigation; supervision; validation; writing – review and editing. **Jyoti Ramnath Kini:** Investigation; supervision; validation; writing – review and editing. **Vatsala Basavaraju Kudurugundi:** Investigation; supervision; validation; writing – review and editing. **Vanishree Ashok:** Investigation; validation. **Chaithra Venkataramana Gowthuvalli:** Investigation; supervision; validation.

## FUNDING INFORMATION

The authors received no specific grant from any funding agency in the public, commercial, or not‐for‐profit sectors.

## CONFLICT OF INTEREST STATEMENT

The authors declare that they have no competing interests.

## ETHICS STATEMENT

All procedures performed in this study involving human participants were in accordance with the 1964 Helinski Declaration and its later amendments. The paper is exempt from ethics committee approval as only one case was reported.

## CONSENT

The patient's guardian gave written consent for their personal and the patient's clinical details and any identifying images published in this study.

## Data Availability

Data sharing is not applicable to this article as no new datasets were generated or analyzed during the current study.
